# Variable setpoint as a relaxing component in physiological control

**DOI:** 10.14814/phy2.13408

**Published:** 2017-09-14

**Authors:** Geir B. Risvoll, Kristian Thorsen, Peter Ruoff, Tormod Drengstig

**Affiliations:** ^1^ Department of Electrical Engineering and Computer Science University of Stavanger Stavanger Norway; ^2^ Centre for Organelle Research University of Stavanger Stavanger Norway

**Keywords:** Integral control, negative feedback, rheostasis, sodium homeostasis, variable setpoint

## Abstract

Setpoints in physiology have been a puzzle for decades, and especially the notion of fixed or variable setpoints have received much attention. In this paper, we show how previously presented homeostatic controller motifs, extended with saturable signaling kinetics, can be described as variable setpoint controllers. The benefit of a variable setpoint controller is that an observed *change* in the concentration of the regulated biochemical species (the controlled variable) is fully characterized, and is not considered a deviation from a fixed setpoint. The variation in this biochemical species originate from variation in the disturbances (the perturbation), and thereby in the biochemical species representing the controller (the manipulated variable). Thus, we define an *operational space* which is spanned out by the combined *high* and *low* levels of the variations in (1) the controlled variable, (2) the manipulated variable, and (3) the perturbation. From this operational space, we investigate *whether* and *how* it imposes constraints on the different motif parameters, in order for the motif to represent a mathematical model of the regulatory system. Further analysis of the controller's ability to compensate for disturbances reveals that a variable setpoint represents a relaxing component for the controller, in that the necessary control action is reduced compared to that of a fixed setpoint controller. Such a relaxing component might serve as an important property from an evolutionary point of view. Finally, we illustrate the principles using the renal sodium and aldosterone regulatory system, where we model the variation in plasma sodium as a function of salt intake. We show that the experimentally observed variations in plasma sodium can be interpreted as a variable setpoint regulatory system.

## Introduction

Setpoints in physiology have been a puzzle for decades, and issues like (1) do setpoints exist? (2) what is the level of the setpoint? (3) is the setpoint fixed or variable? (4) how can the setpoint be mathematically expressed? and (5) what are the possible biochemical mechanisms behind a setpoint? have been extensively discussed (Cram 1983; Nemeth et al. 1986; Koeslag et al. 1997; Mekjavić et al. 1991; Briese 1998; Saunders et al. 1998; Kronzucker et al. 2003; Kurbel et al. 2003; St Clair Gibson et al. 2005; Cabanac 2006). Many of these issues have further been related to the concepts of homeostasis (Cannon 1929; Langley 1973; Cooper 2008), predictive homeostasis (Moore‐Ede 1986), rheostasis (Mrosovsky 1990), and allostasis (Mathison 1995; Sterling et al. 1988; Schulkin 2003; Stumvoll et al. 2003; Sterling 2004).

One of the first attempts to describe what can actually be interpreted as a variable setpoint, was done by Ludwig (1885) when studying the physiological responses to variations in salt intake. Extracts from his work is presented by Bonventre and Leaf (1982b) where they argue for the existence of sodium homeostasis without a fixed setpoint. Prior to this, Hollenberg (1980) described a fixed setpoint for sodium being the sodium level at no‐salt intake. The discussion between Hollenberg on one side and Bonventre and Leaf on the other continued in Hollenberg (1982) and Bonventre and Leaf (1982a).

In the last decades, the notion of a physiological setpoint have repeatedly been revisited, in particular in relation to the concepts of integral feedback control and perfect adaptation (Yi et al. 2000; Saunders et al. 2000; El‐Samad et al. 2002; Ma et al. 2009; Drengstig et al. 2012a; Ang et al. 2013; Somvanshi et al. 2015; Briat et al. 2016). Most of these contributions view the regulatory networks from a control theoretic perspective where a fixed setpoint is the main goal. Common for the “fixed setpoint” approaches are the lack of a framework to include and describe the situation where the controlled variable deviates from the setpoint. An example of such is presented by ourselves (Drengstig et al. 2012a) where we termed this deviation for controller *accuracy*.^1^ The existence of such accuracy measures in physiological controllers have also been found by others. In the work by Ma et al. (2009), they introduced the terms *Sensitivity* and *Precision* to quantify the level of accuracy, whereas Ang and McMillen (2013) use the term *near‐perfect* adaptation for the same. Others again (including ourselves) have also defined such a response for *partial adaptation* (Asthagiri et al. 2000; Drengstig et al. 2008). Each of these different classifications of setpoint deviation indicates that the complexity of physiological regulatory systems exceed (not really surprisingly) the functionality/complexity available in standard control theoretic terminology.

Leaving the search for a fixed setpoint and instead focus on characterizing a variable setpoint, give us the framework to also describe other aspects of physiological control. One such aspect is the assistance provided to the controller from variations in the controlled variable. This assistance represents a relaxing component for the controller as the outcome is reduced control effort, which makes it interesting from an evolutionary point of view. Furthermore, a variable setpoint description shares similarities with *rheostasis* (Mrosovsky 1990), and based on how Mrosovsky (1990) describes this variation, that is, “Change is not a failure of regulation, but an adaptive response, promoting the survival of the animal”, we will in this paper reinvestigate our previously published controller motifs (Drengstig et al. 2012a) from a rheostatic point of view.

### Computational methods

Rate equations were solved symbolically and numerically by using MATLAB/SIMULINK. To make notations simpler, concentrations of compounds are denoted by compound names without square brackets. Concentrations and rate constants are given in arbitrary units (a.u.) if not stated otherwise.

## Controller Motifs With Saturable Signaling Kinetics

**Figure 1:**
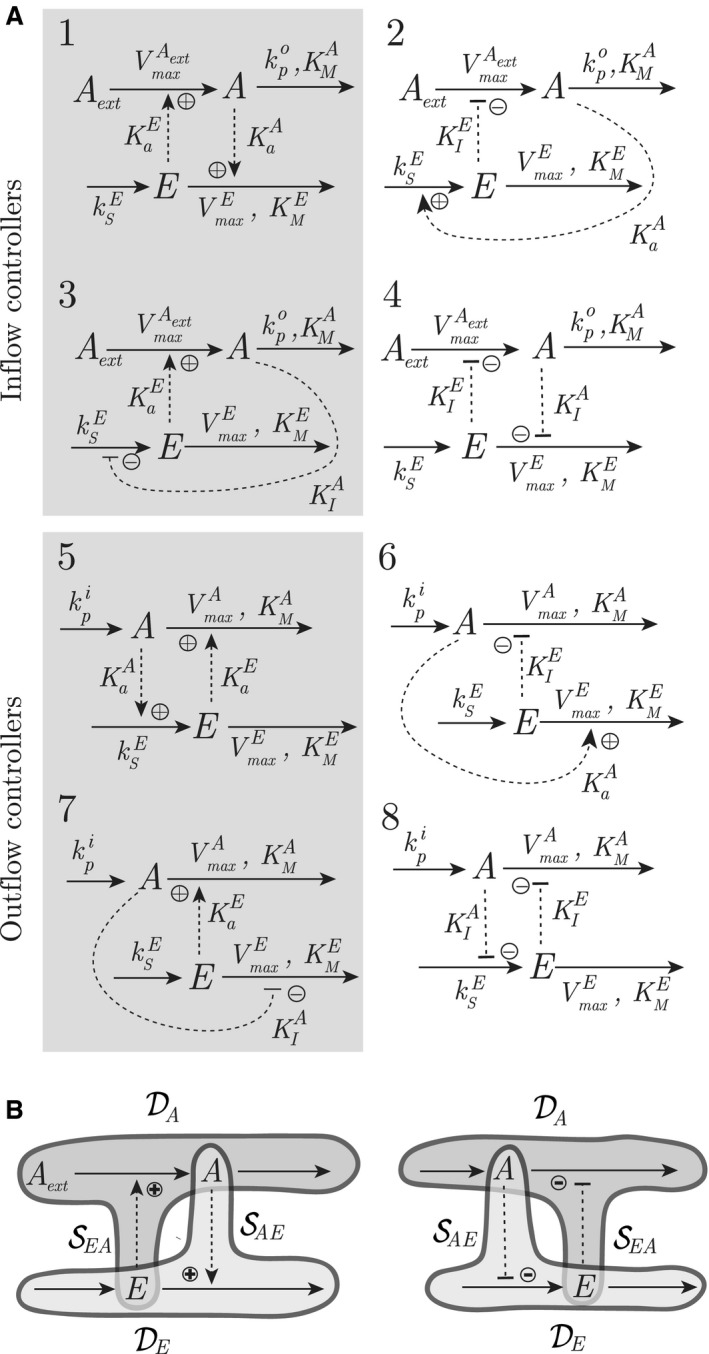
(A) Inflow and outflow controllers with saturable activating action (gray background) or inhibiting action (white background). The controlled species *A* is subject to outflow or inflow perturbation ($k_{p}^{o}$ or $k_{p}^{i}$), where the controller species *E* compensates for this perturbation through *E*‐mediated inflow or outflow of *A*, respectively. The synthesis of *E* is modeled with a rate constant $k_{s}^{E}$, whereas the degradation of *E* is assumed to be a saturable enzymatic reaction with a Michaelis–Menten constant $K_{M}^{E}$. Similar saturable enzymatic reactions are also assumed in the degradation of controlled species *A*. (B) Illustration of how the different parameter sets $D_{A}/S_{EA}$ (dark gray), and $D_{E}/S_{AE}$ (light gray) relate to the different motif parts. The two examples are inflow controller 1 (left) and outflow controller 8 (right).

As a preamble, we present in this section a short summary of previously published homeostatic controller motifs (Drengstig et al. 2012a). These motifs consist of a controlled species *A* and a controller species *E* interacting with each other in different negative feedback configurations. Based on the controller action, these controller motifs are further classified as either inflow or outflow controllers with activating or inhibiting control action, see Figure 1A. The activating signaling kinetics between *A* and *E* in our models (Drengstig et al. 2012a,b; Thorsen et al. 2013) have so far been based on first‐order kinetics, which implies that the controller species *E* in theory can compensate for infinite level of perturbation. This signaling model are in many modeling efforts an adequate simplification (Bocharov et al. 2011; Palumbo et al. 2013), and could as such have been here used to describe the relationship between fixed and variable setpoints. However, as the use of more complex signaling events are in other modeling efforts a better assumption (Korsgaard et al. 2006; Ang et al. 2011; Schaber et al. 2013), we have in this paper extended our controller motifs to include saturable signaling kinetics between *A* and *E*.

To illustrate the saturable signaling kinetics, we refer to Figure 1A, and show the model equations (1) and (2) for inflow controller 1

(1) $$\dot{A}=V_{\max}^{A_{\mathrm{ext}}}\cdot A_{\mathrm{ext}}\cdot \frac{E}{\left(K_{a}^{E}+E\right)}-k_{p}^{o}\cdot \frac{A}{K_{M}^{A}+A}$$

(2) $$\dot{E}=k_{s}^{E}-V_{\max}^{E}\cdot \frac{E}{\left(K_{M}^{E}+E\right)}\cdot \frac{A}{\left(K_{a}^{A}+A\right)}$$

where the kinetics between *A* and *E*, and between *E* and *A*, are characterized by the activation constants $K_{a}^{A}$ and $K_{a}^{E}$, respectively. The variable $k_{p}^{o}$ represents an uncontrolled outflow perturbation, which is compensated by the *E*‐mediated inflow of *A*. $A_{\mathrm{ext}}$ is an external source of *A* generating the compensatory flux opposing $k_{p}^{o}$. The enzymatic degradation of *A* and *E* are modeled as standard Michaelis–Menten expressions.

To organize the different parameters occurring in all of the eight controller motifs, we sort them into the following sets:

$$\begin{array}{c} D_{A}=\left\{V_{\max}^{A_{\mathrm{ext}}},K_{M}^{A},V_{\max}^{A}\right\}\;S_{EA}=\left\{K_{a}^{E},K_{I}^{E}\right\}\\ D_{E}=\left\{k_{s}^{E},V_{\max}^{E},K_{M}^{E}\right\}\;S_{AE}=\left\{K_{a}^{A},K_{I}^{A}\right\} \end{array}$$

where $D_{A}$ is related to the *dynamics* of *A*, $S_{EA}$ is related to the *signaling* from *E* to *A*, $D_{E}$ is related to the *dynamics* of *E*, and $S_{AE}$ is related to the *signaling* from *A* to *E*. The dynamics of *A* and *E* for all eight controller motifs can then be written as

(3) $$\dot{A}=f_{1}\left(A,E,D_{A},S_{EA},k_{p}^{i/o}\right)$$

(4) $$\dot{E}=f_{2}\left(A,E,D_{E},S_{AE}\right)$$

where the functions *f*_1_(·) and *f*_2_(·) are the basis for the analysis shown later. A graphical illustration of this structure is shown in Figure 1B for inflow controller 1 and outflow controller 8.

From control theory, we know that integral action is necessary to keep a controlled variable at a fixed setpoint in the presence of disturbances (Åström et al. 1995). For our previously published controller motifs, zero‐order degradation of the controller species *E* is a necessary condition for the motifs to have integral action (Drengstig et al. 2012a), and based on this condition we developed a procedure to calculate a fixed setpoint (Drengstig et al. 2012a). In short, this procedure use the differential equation for the controller species *E* and assume (1) zero‐order kinetics, that is, $K_{M}^{E}\ll E$ (in practice $K_{M}^{E}\;=\;0$) and (2) steady‐state condition ($\dot{E}\;=\;0$), to determine the steady‐state value of *A*. As this value of *A* is independent of the perturbations, it represents therefore the fixed setpoint $A_{\mathrm{set}}$. The procedure then returns to the original differential equation for *E*, and reorganizes it into a structure similar to the integral control law $\dot{E}\;=\;G_{i}\cdot \left(A_{\mathrm{set}}-A_{\mathrm{meas}}\right)$. Here, $G_{i}$ is the controller gain and $A_{\mathrm{meas}}$ is the measurement or feedback function. However, since $A_{\mathrm{set}}$ is calculated assuming $K_{M}^{E}\;=\;0$, the level of *A* will not adapt to $A_{\mathrm{set}}$, and as mentioned above, we termed this deviation for *accuracy α* (Drengstig et al. 2012a).

## Results and Discussion

Throughout this section, we will use controller motif 1 given by Equations (1) and (2) as an illustrative example. First, we will present the *structural* differences behind the fixed setpoint approach and the new variable setpoint approach. Thereafter, we will give an in depth analysis of the variable setpoint controller.

### The homeostatic view of controller motifs

In this paper, we term the procedure described above for calculating the fixed setpoint (Drengstig et al. 2012a) as the *homeostatic view* approach. Using the procedure on the differential equation for *E* in Equation (2) gives the reorganized equation in Equation (5).

(5) $$\dot{E}=\underset{G_{i}}{\underbrace{\frac{V_{\max}^{E}-k_{s}^{E}}{K_{a}^{A}+A}}}\left(\underset{A_{\mathrm{set}}}{\underbrace{\frac{k_{s}^{E}K_{a}^{A}}{V_{\max}^{E}-k_{s}^{E}}}}-\underset{A_{\mathrm{meas}}}{\underbrace{\left(\frac{V_{\max}^{E}\cdot \frac{E}{K_{M}^{E}+E}-k_{s}^{E}}{V_{\max}^{E}-k_{s}^{E}}\right)\cdot A}}\right)$$

As we see, the expression for the fixed setpoint consists *only* of parameter values from the sets $D_{E}$ and $S_{AE}$. Since this is generally true for all of the eight controller motifs in Figure 1A, the integral control law from the *homeostatic view* can be expressed as

(6) $$\dot{E}=G_{i}\left(D_{E},S_{AE},A,E\right)\cdot \left(A_{set}\left(D_{E},S_{AE}\right)-A_{\mathrm{meas}}\left(D_{E},S_{AE},A,E\right)\right)$$

**Figure 2:**
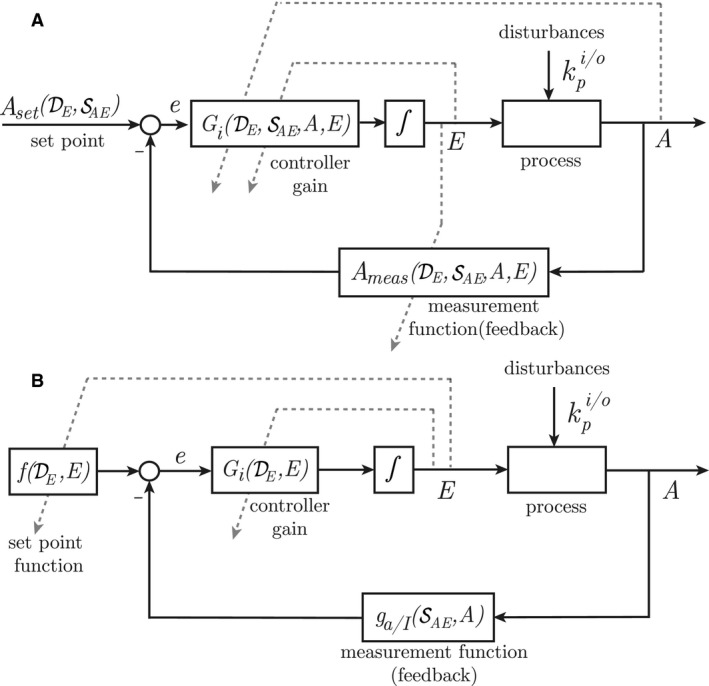
Negative feedback loops for the homeostatic view (A) and the rheostatic view (B) of controller motifs. Functionally there is no difference between solid and dashed lines. Solid lines are used to highlight the well known negative feedback configuration, whereas dashed lines are used to indicate additional functionality which traverse their target to resemble an adjustment. Note that the setpoint calculation in (A) only depends on parameter values, whereas the setpoint in (B) in addition depends on the level of *E*, and hence, becomes a variable setpoint.

The syntax $A_{\mathrm{set}}\left(D_{E},S_{AE}\right)$ indicates that $A_{\mathrm{set}}$ is a function of the parameters in $D_{E}$ and $S_{AE}$. The structure in Equation (6) is schematically illustrated in Figure 2A, which is recognized as a negative feedback loop with integral action, and where the dashed arrows indicate additional information flow in the control loop. From a control theoretic point of view, the information about the level of *A* which is fed back to the controller has similarities with gain scheduling (Åström et al. 1995), which is an adaptive control strategy. On the other hand, the information about the level of *E* fed back to the controller gain and fed forward to the measurement function are not common in control engineering. However, the structure has similarities to Figure 8 in the work of He et al. (2013), where the integral part of the controller is partly represented by a first‐order system.

The structure in Figure 2A gives an intuitive explanation of why deviation from a fixed setpoint occurs, since the information arrow from *E* fed forward to the measurement function $A_{\mathrm{meas}}\left(\cdot \right)$ represents the ratio $E/(K_{M}^{E}+E)$ (see Eq. (5)). In a situation where $K_{M}^{E}\neq 0$, this ratio is less than unity, which implies that the output from the measurement function $A_{\mathrm{meas}}\left(\cdot \right)$ will no longer reflect the level of *A* alone. As the output from $A_{\mathrm{meas}}\left(\cdot \right)$ will become equal to *A*_*set*_(·) (control error *e* = 0), the level of *A* will *not* adapt to $A_{\mathrm{set}}\left(\cdot \right)$. Thus, the deviation from $A_{\mathrm{set}}\left(\cdot \right)$ will change according to the level of *E*.

### The rheostatic view of controller motifs

The idea behind the *rheostatic view* of controller motifs is to describe the regulatory behavior in terms of a variable/rheostatic setpoint. Thus, instead of a fixed setpoint together with a variable deviation, we lump it all into a variable setpoint. In this regard, we use the fact that the deviation depends on the level of *E* as described above. In other words, by reorganizing the differential equation for *E* directly, we find a setpoint which incorporates not only parameters, but also the variable *E*. This is shown in Equation (7) for inflow controller 1 from Equation (2)

(7) $$\dot{E}=\underset{G_{i}}{\underbrace{V_{\max}^{E}\cdot \frac{E}{\left(K_{M}^{E}+E\right)}}}\cdot \left(\underset{f(\cdot )}{\underbrace{\frac{k_{s}^{E}}{V_{\max}^{E}}\cdot \frac{\left(K_{M}^{E}+E\right)}{E}}}-\underset{g_{a}\left(\cdot \right)}{\underbrace{\frac{A}{\left(K_{a}^{A}+A\right)}}}\right)$$

Here, $G_{i}\left(\cdot \right)$ still represents the controller gain, *f*(·) is the rheostatic setpoint *function*, and $g_{a}\left(\cdot \right)$ is the measurement function based on activating signaling kinetics. Motifs with inhibiting signaling from *A* to *E* will in the same way have a measurement function based on inhibiting signaling kinetics *g*_*I*_(·), and hence, a general structure for Equation (7) valid for all eight controller motifs is:

(8) $$\dot{E}=G_{i}\left(D_{E},E\right)\cdot \left(f\left(D_{E},E\right)-g_{a/I}\left(S_{AE},A\right)\right)$$

This new structure is illustrated in Figure 2B, and we argue that this way of looking at the system has several advantages compared to Figure 2A. The most obvious one is that the information flow from the level of *E* to the measurement function is removed, implying that there is no need for any accuracy measures. Instead the information about *E* is fed back to the setpoint, which will vary according to the level of *E*. Since the level of *E* reflects the level of disturbances, the adjustment of the setpoint is, from a physiological point of view, a way to relax the control system. In this context, the signaling kinetics between *E* and *E* is of importance, and we will return to this towards the end of the paper.

### Analysis of the rheostatic controller

Both of the measurement functions $g_{a}\left(S_{AE},A\right)$ and $g_{I}\left(S_{AE},A\right)$ transform the actual level of *A* into a *relative* value between 0 and 1. Consequently, the value of the rheostatic setpoint function *f*(·) must also be a value between 0 and 1, and at steady state, the control error *e* = 0 and

(9) $$f\left(D_{E},E\right)=g_{a/I}\left(S_{AE},A\right)$$

Since the steady‐state level of *A* will *always* be identical to the variable setpoint value, we define the rheostatic setpoint $A_{\mathrm{set}}^{\mathrm{rheo}}$ as $A\;=\;A_{\mathrm{set}}^{\mathrm{rheo}}$. Inserting this into Equation (9) and solving for $A_{\mathrm{set}}^{\mathrm{rheo}}$, we find

(10) $$A_{\mathrm{set}}^{\mathrm{rheo}}=g_{a/I}^{-1}\left(S_{AE},f\left(D_{E},E\right)\right)$$

Similar to Equation (6), we write the setpoint as $A_{\mathrm{set}}^{\mathrm{rheo}}\left(S_{AE},D_{E},E\right)$. We have considered all of the eight controller motifs in Figure 1A from this new viewpoint and derived the symbolic expression for $G_{i}\left(D_{E},E\right)$ and $f(D_{E},E)$ from Equation (8), together with $A_{\mathrm{set}}^{\mathrm{rheo}}\left(S_{AE},D_{E},E\right)$. These are all shown in Table 1. In the following sections, we will analyze different aspects of this new definition of a variable setpoint. We will use that the steady‐state levels of *A*,* E*, and $k_{p}^{i/o}$ are dependent and that they can be organized into combinations of *high* and/or *low* steady‐state levels. We recognize that these high and low levels can be related to what Cannon (1929); termed *physiological range*. Since our definition of a variable setpoint depends on several of the motif parameters, we will also analyze how the combinations of steady‐state levels relates to the different motif parameters.

**Table 1:** Expressions for $G_{i}\left(D_{E},E\right)$, $f(D_{E},E)$, and $A_{\mathrm{set}}^{\mathrm{rheo}}\left(S_{AE},D_{E},E\right)$ for all eight controller motifs, together with the corresponding measurement function $g_{a/I}\left(S_{AE},A\right)$

Motif	$G_{i}\left(D_{E},E\right)$	$f(D_{E},E)$	$g_{a/I}\left(S_{AE},A\right)$	$A_{\mathrm{set}}^{\mathrm{rheo}}\left(S_{AE},D_{E},E\right)$
1, 6	$V_{\max}^{E}\cdot \frac{E}{\left(K_{M}^{E}+E\right)}$	$\frac{k_{s}^{E}}{V_{\max}^{E}}\cdot \frac{\left(K_{M}^{E}+E\right)}{E}$	$\frac{A}{K_{a}^{A}+A}$	$\frac{K_{a}^{A}\cdot k_{s}^{E}\cdot \left(K_{M}^{E}+E\right)}{E\cdot \left(V_{\max}^{E}-k_{s}^{E}\right)-K_{M}^{E}\cdot k_{s}^{E}}$
2, 5	$-k_{s}^{E}$	$\frac{V_{\max}^{E}}{k_{s}^{E}}\cdot \frac{E}{\left(K_{M}^{E}+E\right)}$	$\frac{A}{K_{a}^{A}+A}$	$\frac{K_{a}^{A}\cdot V_{\max}^{E}\cdot E}{E\cdot \left(k_{s}^{E}-V_{\max}^{E}\right)+K_{M}^{E}\cdot k_{s}^{E}}$
3, 8	$-k_{s}^{E}$	$\frac{V_{\max}^{E}}{k_{s}^{E}}\cdot \frac{E}{\left(K_{M}^{E}+E\right)}$	$\frac{K_{I}^{A}}{K_{I}^{A}+A}$	$\frac{K_{I}^{A}\cdot k_{s}^{E}\cdot \left(K_{M}^{E}+E\right)}{E\cdot V_{\max}^{E}}-K_{I}^{A}$
4, 7	$V_{\max}^{E}\cdot \frac{E}{\left(K_{M}^{E}+E\right)}$	$\frac{k_{s}^{E}}{V_{\max}^{E}}\cdot \frac{\left(K_{M}^{E}+E\right)}{E}$	$\frac{K_{I}^{A}}{K_{I}^{A}+A}$	$\frac{K_{I}^{A}\cdot V_{\max}^{E}\cdot E}{k_{s}^{E}\cdot \left(K_{M}^{E}+E\right)}-K_{I}^{A}$

In the literature, we find examples where the steady‐state regulatory behavior can be organized into such high and/or low level combinations, for example, plasma sodium levels in relation to aldosterone and salt intake (Laragh et al. 1957), or blood glucose levels in relation to insulin and food intake (Topp et al. 2007).

### Steady‐state trajectory

**Figure 3:**
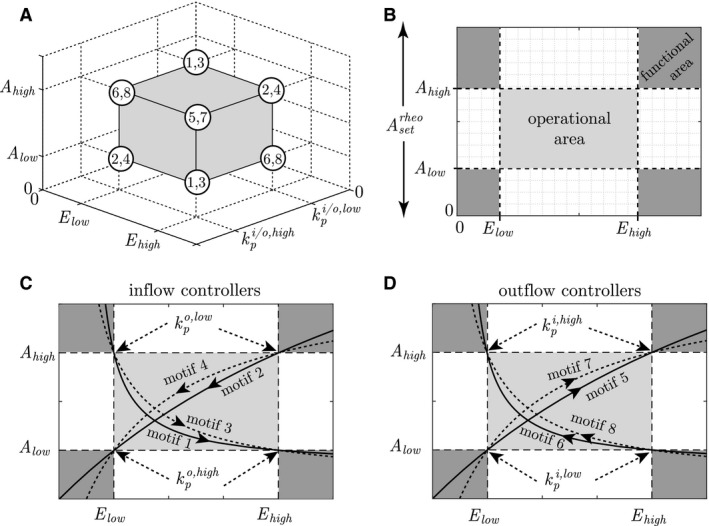
Visualization of the operational space/area and functional area of all eight controller motifs. (A) Operational space spanned out by the high and/or low levels of *A*,* E* and $k_{p}^{i/o}$, where the corners are indexed with a number corresponding to the controller motifs in Figure 1A. A corner represents a combination of steady‐state high/low levels for that motif, and is therefore a location where the steady‐state trajectory by definition goes through. Motifs 5 and 7 go through the hidden lower corner in the back. (B) Projection of the operational space into an operational area (light gray) and functional areas (dark gray) as a function of *A* and *E only*. (C, D) Illustration of how the steady‐state trajectory of the different controller motifs traverse the operational and functional areas. The illustration shows typical behavior and gives a qualitatively description of each motif. The corresponding perturbation levels are indicated in the transition from operational to functional areas, and the arrows on the trajectories indicate the direction of movement when $k_{p}^{i/o}$ increases.

The above‐mentioned dependencies between *A*,* E* and $k_{p}^{i/o}$ define, what we call, an *operational space*, see Figure 3A. This is a three‐dimensional representation of the space spanned out by the combinations of high and/or low levels of *A*,* E* and $k_{p}^{i/o}$. The corners of the cube in Figure 3A represent the combinations of the high/low levels where the steady‐state trajectory of the different motifs go through, and the numbers in the corners correspond to the motifs numbers in Figure 1A. The different pathways through the cube illustrate two properties. First, it reveals the kind of controller, that is, inflow or outflow. This is identified by considering the level of *A* at $k_{p}^{i/o,\mathrm{high}}$. If *A* = *A*_low_, then it is an inflow controller since an outflow perturbation will drag the *A*‐level down. Similarly, if *A* = *A*_high_ at $k_{p}^{i/o,\mathrm{high}}$, then it is an outflow controller since an inflow perturbation will increase the level of *A*. Secondly, it tells us whether there is activating or inhibiting signaling from *E* to the compensatory flow of *A*. This is identified by considering the level of *E* at $k_{p}^{i/o,high}$. If *E* = *E*_high_, then it is an activating controller since $k_{p}^{i/o,\mathrm{high}}$ will be compensated by a high level of *E*. Similarly, if *E* = *E*_low_ at $k_{p}^{i/o,high}$, then it is an inhibiting controller since $k_{p}^{i/o,\mathrm{high}}$ will be compensated by a low level of *E*. Thus, one way to use such an operational space is to foresee *structural* information about the underlying regulatory mechanism based on reported and/or experimentally measured steady‐state values of *A*,* E* and $k_{p}^{i/o}$.

The operational space can be further projected into an operational *area* as a function of *A* and *E* only, see Figure 3B. This enables us to illustrate that the controllers are also able to operate outside the operational area, although exceeding the specified combinations of high/low levels. These additional areas are termed *functional areas*. From a physiological point of view, the transition into a functional area might as well initiate other control mechanisms to bring the organism back into the operational area again, but such mechanisms are not considered in this paper.

The qualitative behavior of the steady‐state trajectories through the operational and functional areas of the eight controller motifs are shown in Figure 3C and D. The arrows on the trajectories indicate the direction of movement when $k_{p}^{i/o}$ increases from $k_{p}^{i/o,\mathrm{low}}$ to $k_{p}^{i/o,\mathrm{high}}$.

As our goal is to make mathematical models able to fit steady‐state levels of *A*,* E*, and $k_{p}^{i/o}$ in terms of a variable setpoint regulatory mechanism, the model behavior depends heavily on model parameters. We will therefore in the following two sections investigate whether and how the defined operational space/area impose constraints on the different motif parameters. In this context, we define the difference between the highest and the lowest level of a variable, e.g. *A*_high_−*A*_low_, as the *range* in that variable. Furthermore, since the saturable signaling kinetics represents a non‐linear mapping of concentration levels into a relative measure, we focus in particular on constraints imposed on the activation and inhibition constants in $S_{EA}$ and $S_{AE}$.

### Imposed constraints on the parameters in $D_{A}$ and $S_{EA}$

We start with the two parameter sets $D_{A}$ and $S_{EA}$ related to the dynamics of *A* and the signaling from *E* to *A*, respectively, and the analysis is therefore based on the steady‐state version of the generalized differential equation of *A* given in Equation (3). By inserting each of the two relevant combinations of high and low levels of *A*,* E* and $k_{p}^{i/o}$, we get a system of two equations and three unknowns ($V_{\max}^{A_{\mathrm{ext}}}$/$V_{\max}^{A}$, $K_{M}^{A}$ and $K_{a}^{E}$/$K_{I}^{E}$). This is shown in Equations (11) and (12) for inflow controller 1 in Equation (1), where we have inserted the combinations representing the corners of the cube in Figure 3A.

(11) $$f_{1}\left(A_{\mathrm{low}},E_{\mathrm{high}},k_{p}^{o,\mathrm{high}},V_{\max}^{A_{\mathrm{ext}}},K_{a}^{E},K_{M}^{A}\right)=0$$

(12) $$f_{1}\left(A_{\mathrm{high}},E_{\mathrm{low}},k_{p}^{o,\mathrm{low}},V_{\max}^{A_{\mathrm{ext}}},K_{a}^{E},K_{M}^{A}\right)=0$$

As the system is underspecified, and because we are particularly interested in the signaling kinetics, we solve the equations with respect to $K_{M}^{A}$ and $V_{\max}^{A_{\mathrm{ext}}}$. These two parameters will then be a function of $K_{a}^{E}$ and the operational space, and thus, in order to obtain positive and real values for $K_{M}^{A}$ and $V_{\max}^{A_{\mathrm{ext}}}$, we identify constraints on $K_{a}^{E}$ as a function of the operational space.

As a general result for all of the eight controller motifs, we identify as parts of the solutions the following two expressions

(13) $$\beta_{1}=\frac{A_{\mathrm{high}}\cdot E_{\mathrm{high}}\cdot k_{p}^{i/o,\mathrm{low}}-A_{\mathrm{low}}\cdot E_{\mathrm{low}}\cdot k_{p}^{i/o,\mathrm{high}}}{A_{\mathrm{low}}\cdot k_{p}^{i/o,\mathrm{high}}-A_{\mathrm{high}}\cdot k_{p}^{i/o,\mathrm{low}}}=\frac{\beta_{1,\mathrm{num}}}{\beta_{1,\mathrm{denom}}}$$

(14) $$\beta_{2}=\frac{E_{\mathrm{high}}\cdot k_{p}^{i/o,\mathrm{low}}-E_{\mathrm{low}}\cdot k_{p}^{i/o,\mathrm{high}}}{k_{p}^{i/o,\mathrm{high}}-k_{p}^{i/o,\mathrm{low}}}=\frac{\beta_{2,\mathrm{num}}}{\beta_{2,\mathrm{denom}}}$$

Note that *β*_1_ takes the entire operational space into consideration, whereas *β*_2_ only considers the ranges in *E* and $k_{p}^{i/o}$, and that both the numerators *β*_1,num_ and *β*_2,num_, and the denominator *β*_1,denom_, can be either positive or negative. Based on the signs of *β*_1,num_, *β*_2,num_, and *β*_1,denom_, Table 2 summarizes the constraints imposed on $K_{a}^{E}$/$K_{I}^{E}$ in order for the steady‐state trajectory of *A*,* E* and $k_{p}^{i/o}$ to go through the corners of the operational area. From Table 2, we see that the sign of *β*_1,num_ determines whether there is a solution or not. If *β*_1,num_ is positive, the four possible combinations of the signs of *β*_1,denom_ and *β*_2,num_ determines the conditions on $K_{a}^{E}$ and $K_{I}^{E}$. We note also that if either *β*_1,denom_ or *β*_2,num_ is negative, then the respective *β*_1_ and *β*_2_ is not a part of the condition. When *β*_1,num_ is negative, it can easily be shown from Equations (13) and (14) that there is only one possible sign combination of *β*_1,denom_ and *β*_2,num_, and for this combination, there is no solution to either $K_{a}^{E}$ or $K_{I}^{E}$.

**Table 2:** Constraints imposed on the parameters $K_{a}^{E}$ and $K_{I}^{E}$, as a function of the sign of *β*_1,num_, *β*_2,num_, and *β*_1,denom_ from Equations (13) and (14)

*β*_1,num_	*β*_2,num_	*β*_1,denom_	$K_{a}^{E}$	$K_{I}^{E}$
−	−	+	no solution	no solution
+	−	−	$K_{a}^{E}>0$	$K_{I}^{E}>0$
+	−	+	$K_{a}^{E}>\frac{E_{\mathrm{high}}\cdot E_{\mathrm{low}}}{\beta_{1}}$	$K_{I}^{E}<\beta_{1}$
+	+	−	$K_{a}^{E}<\frac{E_{\mathrm{high}}\cdot E_{\mathrm{low}}}{\beta_{2}}$	$K_{I}^{E}>\beta_{2}$
+	+	+	$\frac{E_{\mathrm{high}}\cdot E_{\mathrm{low}}}{\beta_{1}}<K_{a}^{E}<\frac{E_{\mathrm{high}}\cdot E_{\mathrm{low}}}{\beta_{2}}$	$\beta_{2}<K_{I}^{E}<\beta_{1}$

So, what is the effect of selecting an arbitrary value for $K_{a}^{E}$/$K_{I}^{E}$ satisfying the conditions in Table. 2? Well, even though the high/low levels of *A* and *E* representing the corners of the operational area are still the same, the steady‐state trajectory *inside* the operational area is slightly altered. However, the largest effect is found in the *dynamic* behavior of the controller motifs. Thus, given time series measurements of *A*,* E* and $k_{p}^{i/o}$ would provide us with data to perform parameter estimation (Isermann et al. 1992). This is, however, not a topic in this paper as we here focus on the steady‐state behavior.

**Figure 4:**
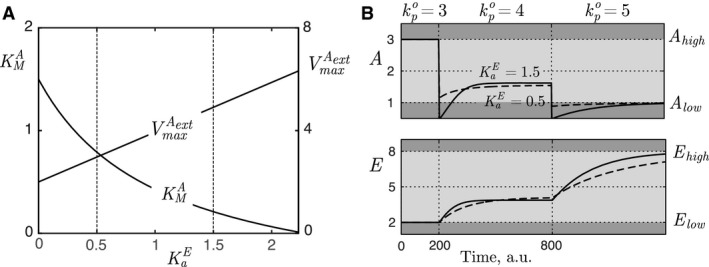
(A) $K_{M}^{A}$ and $V_{\max}^{A_{\mathrm{ext}}}$ (from parameter set $D_{A}$) as a function of $K_{a}^{E}$ (from parameter set $S_{EA}$) for inflow controller 1 in Equations (1) and (2). The operational space is specified as follows: *A*_low_ = 1, *A*_high_ = 3, *E*_low_ = 2, *E*_high_ = 8, $k_{p}^{o,\mathrm{low}}\;=\;3$, and $k_{p}^{o,\mathrm{high}}\;=\;5$. The dashed vertical lines correspond to parameter selection used in (B). (B) Responses in *A* and *E* for a stepwise increase in $k_{p}^{o}$ from $k_{p}^{o,\mathrm{low}}$ = 3–4 at time *t* = 200 a.u., and further increased to $k_{p}^{o,\mathrm{high}}\;=\;5$ at time *t* = 800 a.u. It illustrates that increased $K_{a}^{E}$ influences mostly the dynamics of *A*. Dark and light gray represent functional and operational areas, respectively. Parameter values for solid line: $K_{a}^{E}\;=\;1.5$, $V_{\max}^{A_{ext}}\;=\;5$ and $K_{M}^{A}\;=\;0.19$. Parameter values for dashed line: $K_{a}^{E}\;=\;0.5$, $V_{\max}^{A_{\mathrm{ext}}}\;=\;3$ and $K_{M}^{A}\;=\;0.77$. For both simulations the other parameter values are as follows: $K_{a}^{A}\;=\;8$, $k_{s}^{E}\;=\;0.028$, $K_{M}^{E}\;=\;7.52$, and $V_{\max}^{E}\;=\;0.5$, see the next section.

To illustrate the principles, we use controller motif 1 in Equations (1) and (2), where we assume that the following values are found experimentally and are considered to represent the operational space; *A*_low_ = 1, *A*_high_ = 3, *E*_low_ = 2, *E*_high_ = 8, $k_{p}^{o,\mathrm{low}}\;=\;3$, and $k_{p}^{o,\mathrm{high}}\;=\;5$. Inserting these values into Equations (13) and (14) reveals that *β*_1,denom_ is negative, and that $K_{a}^{E}<2.28$. This is shown in Figure 4A, where $K_{M}^{A}$ and $V_{\max}^{A_{\mathrm{ext}}}$ from the parameter set $D_{A}$ is presented as a function of $K_{a}^{E}$ from the parameter set $S_{EA}$. We observe that $K_{M}^{A}$ becomes negative for $K_{a}^{E}>2.28$.

The effect of selecting different values for $K_{a}^{E}$ within the available range in Figure 4A (and thereby other combinations of $K_{M}^{A}$ and $V_{\max}^{A_{\mathrm{ext}}}$), is found in the dynamic behavior as shown in Figure 4B. We note that the dynamic properties of *A*, especially the level of overshoot, is highly influenced by the level of $K_{a}^{E}$. Note, however, that the steady‐state level of *A* and *E inside* the functional area are slightly altered, implying that the path through the operational space varies as a function of parameter values. Similar results are obtained in the analysis of the outflow controllers.

### Imposed constraints on parameters in $D_{E}$ and $S_{AE}$

Moving on to the parameters in the sets $D_{E}$ and $S_{AE}$ related to the dynamics of *E* and the signaling from *A* to *E*, respectively, it is sufficient to focus on the operational *area* shown in Figure 3B. The reason for this is that the perturbation is not a part of the differential equation of *E*. Similar to the previous section, we focus also here in particular on conditions on the signaling kinetic parameters $K_{a}^{A}/K_{I}^{A}$ in $S_{AE}$.

In general, the parameters $k_{s}^{E}$ and $V_{\max}^{E}$ in $D_{E}$ are related to the dynamic properties of the controller motifs, for example, overshoot and rise time after a step in the disturbance. The explanation behind this is that one of these two parameters always constitute the controller gain $G_{i}$ (see Table 1). Furthermore, both of the parameters are also always part of the rheostatic setpoint. Thus, if the controller gain increases by, for example, increasing the synthesis rate of *E*, then the degradation rate of *E* must also increase in order to maintain the rheostatic setpoint. This implies that these two parameters are dependent, and we take advantage of this in the analysis.

The analysis is based on the steady‐state version of the generalized differential equation of *E* given in Equation (4). By inserting each of the two relevant combinations of high and low levels of *A* and *E* into this equation, we get also here a system of two equations and three unknowns ($k_{s}^{E}/V_{\max}^{E}$, $K_{M}^{E}$, and $K_{a}^{A}$/$K_{I}^{A}$). This is shown in Equations (15) and (16) for inflow controller 1 in Equation (2).

(15) $$f_{2}\left(A_{\mathrm{high}},E_{\mathrm{low}},k_{s}^{E},V_{\max}^{E},K_{M}^{E},K_{a}^{A}\right)=0$$

(16) $$f_{2}\left(A_{\mathrm{low}},E_{\mathrm{high}},k_{s}^{E},V_{\max}^{E},K_{M}^{E},K_{a}^{A}\right)=0$$

Similar to the previous section, we solve for $K_{M}^{E}$ and the ratio of the dependent parameters $k_{s}^{E}/V_{\max}^{E}$, and find that the solutions depend on $K_{a}^{A}$ and the operational area. As a general result for all eight controller motifs, we find the following constraints on $K_{a}^{A}$ and $K_{I}^{A}$:

(17) $$K_{a}^{A}<\left\{\begin{array}{cc} \infty & \text{if}\;A_{\mathrm{high}}\cdot E_{\mathrm{low}}-A_{\mathrm{low}}\cdot E_{\mathrm{high}}<0\\ \frac{A_{\mathrm{high}}\cdot A_{\mathrm{low}}\cdot \left(E_{\mathrm{high}}-E_{\mathrm{low}}\right)}{A_{\mathrm{high}}\cdot E_{\mathrm{low}}-A_{\mathrm{low}}\cdot E_{\mathrm{high}}} & \text{otherwise} \end{array}\right.$$

(18) $$K_{I}^{A}>\left\{\begin{array}{cc} 0 & \text{if}\;A_{\mathrm{high}}\cdot E_{\mathrm{low}}-A_{\mathrm{low}}\cdot E_{\mathrm{high}}<0\\ \frac{A_{\mathrm{high}}\cdot E_{\mathrm{low}}-A_{\mathrm{low}}\cdot E_{\mathrm{high}}}{E_{\mathrm{high}}-E_{\mathrm{low}}} & \text{otherwise} \end{array}\right.$$

From the conditional expressions in Equations (17) and (18), we note that there are no constraints on $K_{a}^{A}$ or $K_{I}^{A}$ if

(19) $$\frac{E_{\mathrm{high}}}{E_{\mathrm{low}}}>\frac{A_{\mathrm{high}}}{A_{\mathrm{low}}}$$

This means that if the variability in *A* is too large or the corresponding variability in *E* is too small, the controller is not able to bring the system through the specified high/low levels, that is, the operational area, without imposing constraints on $K_{a}^{A}$ or $K_{I}^{A}$.

**Figure 5:**
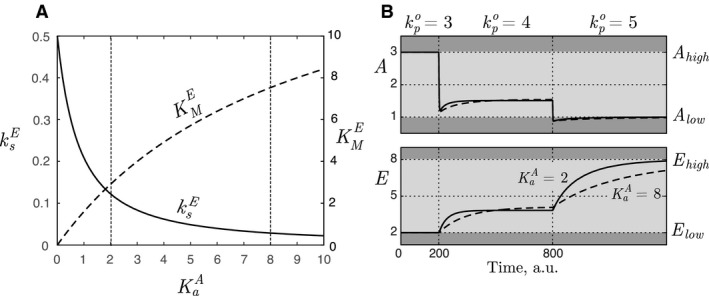
The relationship between the parameters in $D_{E}$ as a function of the parameters in $S_{AE}$. (A) $K_{M}^{E}$ and $k_{s}^{E}$ as a function of $K_{a}^{A}$ for inflow controller 1 in Equations (1) and (2). The dependent parameter $V_{\max}^{E}$ (being the controller gain) is specified as $V_{\max}^{E}\;=\;0.5$. The operational area is the same as in Figure 4, that is, *A*_low_ = 1, *A*_high_ = 3, *E*_low_ = 2 and *E*_high_ = 8. The two vertical lines are related to the results in B. (B) Responses in *A* and *E* for the system in A for a stepwise increase in $k_{p}^{o}$ from $k_{p}^{o,\mathrm{low}}$ = 3–4 at time *t* = 200 a.u., and further increased to $k_{p}^{o,high}\;=\;5$ at time *t* = 800 a.u. It illustrates that increased $K_{a}^{A}$ influences mostly the dynamics of *E*. Dark and light gray represent functional and operational areas, respectively. The dashed curve is the same as the dashed curve in Figure 4B. Parameter values for solid line: $K_{a}^{A}\;=\;2$, $k_{s}^{E}\;=\;0.12$ and $K_{M}^{E}\;=\;2.91$. Parameter values for dashed line: $K_{a}^{A}\;=\;8$, $k_{s}^{E}\;=\;0.028$ and $K_{M}^{E}\;=\;7.52$. For both simulations the other parameter values are as follows: $V_{\max}^{E}\;=\;0.5$, $K_{a}^{E}\;=\;0.5$, $V_{\max}^{A_{\mathrm{ext}}}\;=\;3$, and $K_{M}^{A}\;=\;0.77$.

So, what is the effect of selecting an arbitrary value for $K_{a}^{A}$ or $K_{I}^{A}$ if the condition in Equation (19) is fulfilled? Similar to in the previous section, it alters the solution to the related parameters $k_{s}^{E}/V_{\max}^{E}$ and $K_{M}^{E}$. This is illustrated in Figure 5A for inflow controller 1 in Equations (1) and (2), where we have specified the controller gain $G_{i}$ to $V_{\max}^{E}\;=\;0.5$ (see Table 1). The largest effect of varying $K_{a}^{A}$ (and thereby also $k_{s}^{E}$ and $K_{M}^{E}$) within the available range is also here found in the dynamic behavior. This is shown in Figure 5B for stepwise increases in the outflow perturbation, where an increased $K_{a}^{A}$ results in slower response in *E*.

### The relaxing impact of a variable setpoint

In realistic models of biochemical systems/physiological processes with (1) saturable signaling kinetics and (2) saturable reaction kinetics, it is a challenge to have an intuitive understanding of how a controller motif is able to compensate for large variations in the perturbation. The comprehensional difficulty lies in the fact that the controller's maximum impact on the compensatory flux of *A* is limited to 1, and the maximum dependence on the substrate species concentration is also only 1 (through the Michaelis–Menten relationship). As we will show, the key to understand this puzzle is found in the *ratios* of signaling values and Michaelis–Menten expressions at high and low levels of *E* and *A*, respectively, and from this we identify a relaxing component in physiological control.

Let us first consider how the manipulated variable *E* through the saturable signaling kinetics is able to compensate for large variations in $k_{p}^{i/o}$. Since both the activating and the inhibiting functions from *E* to *A* are structurally similar to the measurement functions $g_{a/I}\left(S_{AE},A\right)$ defined in Table 1, we reuse the function names as $g_{a/I}\left(S_{EA},E\right)$, where

(20) $$g_{a}\left(K_{a}^{E},E\right)=\frac{E}{K_{a}^{E}+E}$$

(21) $$g_{I}\left(K_{I}^{E},E\right)=\frac{K_{I}^{E}}{K_{I}^{E}+E}$$

**Figure 6:**
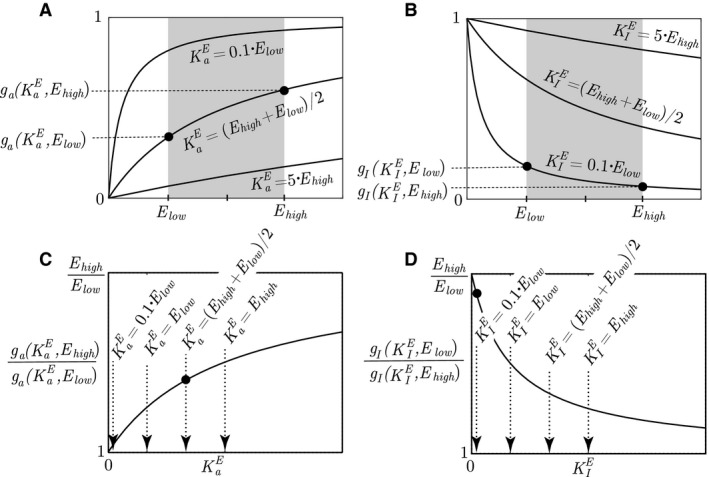
(A) The functional value of the activating signaling kinetics in Equation (20) as a function of $K_{a}^{E}$ and *E*. The values of $K_{a}^{E}$ for the three lines are $K_{a}^{E}\;=\;0.1\cdot E_{\mathrm{low}}$, $K_{a}^{E}\;=\;\left(E_{\mathrm{low}}+E_{\mathrm{high}}\right)/2$, and $K_{a}^{E}\;=\;5\cdot E_{\mathrm{high}}$. The black dots correspond to the readings on the ordinate axis, which is linked to the black dot in C. (B) The functional value of the inhibiting signaling kinetics in Equation (21) as a function of $K_{I}^{E}$ and *E*. The values of $K_{I}^{E}$ for the three lines are $K_{I}^{E}\;=\;0.1\cdot E_{\mathrm{low}}$, $K_{I}^{E}\;=\;\left(E_{\mathrm{low}}+E_{\mathrm{high}}\right)/2$, and $K_{I}^{E}\;=\;5\cdot E_{\mathrm{high}}$. The black dots correspond to the readings on the ordinate axis, which is linked to the black dot in D. (C) The ratio of the highest to lowest value of $g_{a}\left(K_{a}^{E},E\right)$, corresponding to Equation (22). The black dot represents the amplification performed by the controller going from *E*_low_ to *E*_high_ in (A), using $K_{a}^{E}\;=\;\left(E_{\mathrm{low}}+E_{\mathrm{high}}\right)/2$. (D) The ratio of the highest to lowest value of $g_{I}\left(K_{I}^{E},E\right)$, corresponding to Equation (23). The black dot represents the amplification performed by the controller going from *E*_high_ to *E*_low_ in B, using $K_{I}^{E}\;=\;0.1\cdot E_{\mathrm{low}}$

The functional values of Equations (20) and (21) as a function of *E* and different values of $K_{a}^{E}$ or $K_{I}^{E}$ are shown in Figures 6A and B, and we note that the maximum difference in the signaling value is 1. As two examples, we have indicated the functional values of $g_{a}\left(K_{a}^{E},E\right)$ and $g_{I}\left(K_{a}^{E},E\right)$ at *E*_low_ and *E*_high_ for $K_{a}^{E}\;=\;\left(E_{\mathrm{low}}+E_{\mathrm{high}}\right)/2$ and $K_{I}^{E}\;=\;0.1\cdot E_{\mathrm{low}}$, respectively. The change in the functional values when going from *E*_low_ to *E*_high_ (activating controller in Figure 6A), or from *E*_high_ to *E*_low_ (inhibiting controller in Figure 6B), are rather small. However, as the manipulated variable *E* varies between *E*_high_ and *E*_low_, the controller performance is not characterized in the range between the functional values, but rather in the ratio. The reason for this is that the *relative* change in the functional value represents the control signal amplification.

For the activating and inhibiting controllers, these ratios are given in Equations (22) and (23).

(22) $$\frac{g_{a}\left(K_{a}^{E},E_{\mathrm{high}}\right)}{g_{a}\left(K_{a}^{E},E_{\mathrm{low}}\right)}$$

(23) $$\frac{g_{I}\left(K_{I}^{E},E_{\mathrm{low}}\right)}{g_{I}\left(K_{I}^{E},E_{\mathrm{high}}\right)}$$

and illustrated in Figures 6C and D. Interesting, we find the largest amplification when the functional values of $g_{a}\left(K_{a}^{E},E\right)$ and $g_{I}\left(K_{I}^{E},E\right)$ are at their smallest. Thus, the maximum amplification value of *E*_high_/*E*_low_ is obtained when $K_{a}^{E}\to \infty$ or $K_{I}^{E}\to 0$, and this rather contradictory result is the key to the puzzle.

To illustrate how these ratios imply that a variable setpoint represents a relaxing component, we consider again inflow controller 1 in Equations (1) and (2). Since this is an activating controller, the controller species *E* will be at *E*_high_ when the disturbance is at $k_{p}^{o,high}$ (*E*_low_ and $k_{p}^{o,low}$ are similarly related), and the controller amplification/ratio shown in Figure 6C must therefore be related to the *ratio* of the perturbation rate constants. Thus, from the quotient between the steady‐state relationships in Equations (11) and (12), we identify this ratio as

(24) $$\frac{k_{p}^{o,\mathrm{high}}}{k_{p}^{o,\mathrm{low}}}=\frac{E_{\mathrm{high}}\cdot A_{\mathrm{high}}\cdot \left(K_{a}^{E}+E_{\mathrm{low}}\right)\cdot \left(K_{M}^{A}+A_{\mathrm{low}}\right)}{E_{\mathrm{low}}\cdot A_{\mathrm{low}}\cdot \left(K_{a}^{E}+E_{\mathrm{high}}\right)\cdot \left(K_{M}^{A}+A_{\mathrm{high}}\right)}$$

(25) $$=\frac{g_{a}\left(K_{a}^{E},E_{\mathrm{high}}\right)}{g_{a}\left(K_{a}^{E},E_{\mathrm{low}}\right)}\cdot \frac{A_{\mathrm{high}}\cdot \left(K_{M}^{A}+A_{\mathrm{low}}\right)}{A_{\mathrm{low}}\cdot \left(K_{M}^{A}+A_{\mathrm{high}}\right)}$$

Using further that the Michaelis–Menten expression is structurally similar to the activating signaling kinetics in Equation (20), the ratio in Equation (25) can be written as

(26) kpo,highkpo,low=ga(KaE,Ehigh)ga(KaE,Elow)⏟ratiofromcontroller·ga(KMA,Ahigh)ga(KMA,Alow)⏟relaxingfactor

Here, we identify the last part as the relaxing factor, since that ratio has a value larger than 1 (similar to Eq. (22)). This implies that the controller is assisted from variations in *A* in its task of compensating for the disturbances, that is, the variations in *A* reduces the necessary amplification in the controller output obtained by increasing textit*E* from *E*_low_ to *E*_high_. It is here worth repeating that the variations in *A* represents the rheostatic setpoint $A_{\mathrm{set}}^{\mathrm{rheo}}$.

To illustrated this concept using a familiar process, consider a tank of water with a level controller manipulating a valve in the outlet pipe. The inflow of water into the tank is considered a disturbance. If the inflow perturbation increases, a rheostatic controller with a variable setpoint would let the water level in the tank increase in order to take advantage of the increased hydrostatic pressure. Compared with a standard controller with a fixed setpoint, the necessary effort represented by changes in the controlled variable, is for a rheostatic controller reduced since the increased hydrostatic pressure increases the outflow in itself. As long as the increased water level is neither a safety issue nor a product quality issue, it is beneficial with respect to wear and tear of the equipment. Or in the context of physiology, Mrosovsky's statement (Mrosovsky 1990) is worth repeating: “Change is not a failure of regulation, but an adaptive response, promoting the survival of the animal”.

### Illustrating the principles

We will illustrate the principles presented here using the renal plasma sodium and aldosterone regulatory system (Hollenberg 1982). In this context, the salt intake is considered a disturbance for the regulatory system. We will show that the described variation in steady‐state plasma sodium concentration is in accordance with a variable setpoint description for sodium. Note that the model we make is a very simple representation of all the physiological events occurring in body sodium regulation, but the example demonstrates how such regulatory systems can be abstracted into a two‐component controller motif representation. Examples of other physiological processes modeled in a similar way include blood glucose regulation (Bolie 1961; Cobelli et al. 1987); and calcium oscillations (Sneyd et al. 2004), to mention a few.

One of the important hormones in the regulation of body sodium is aldosterone, which is part of the renin–angiotensin–aldosterone system (RAAS) (Garrett et al. 2012). When plasma sodium concentration is, for example, low, the function of the RAAS is essentially to initiate a series of intermediate steps resulting in the synthesis of the peptide angiotensin, which in turn stimulates the secretion of aldosterone from the adrenal cortex. Aldosterone causes the kidney to increase the reabsorption of sodium ions, thereby reducing the urinary sodium excretion (Garrett et al. 2012). Altogether, the overall function of the RAAS can be summarized and simplified as follows:

- At high salt intake ($k_{p}^{i,\mathrm{high}}$), the sodium level (represented as the controlled variable *A*) is high, and thus, the regulatory system can be represented as an outflow controller.
- At high salt intake, the aldosterone level (represented as the manipulated variable *E*) is low. This implies that sodium reabsorption decreases, and the net sodium excretion is increased. Thus, the signaling from aldosterone to the compensatory sodium outflow is based on inhibiting kinetics.

**Figure 7:**
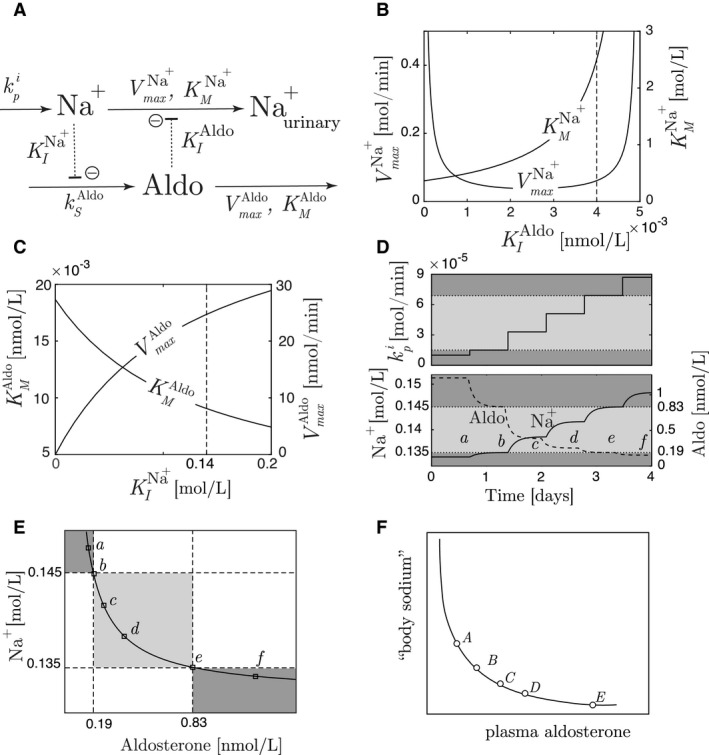
(A) The renal sodium/aldosterone system modeled as an outflow controller 8. (B) The solution to the parameters $K_{M}^{{\mathrm{Na}}^{+}}$ and $V_{\max}^{{\mathrm{Na}}^{+}}$ as a function of $K_{I}^{\mathrm{Aldo}}$. The vertical line corresponds to the selected value of $K_{I}^{\mathrm{Aldo}}\;=\;4\cdot 10^{-3}$ nmol/L. (C) The solution to the parameters $K_{M}^{\mathrm{Aldo}}$ and $V_{\max}^{\mathrm{Aldo}}$ as a function of $K_{I}^{{\mathrm{Na}}^{+}}$ for a given $k_{s}^{\mathrm{Aldo}}\;=\;0.48$ nmol/min. The vertical line corresponds to the selected value of $K_{I}^{{\mathrm{Na}}^{+}}\;=\;0.14$ mol/L. (D) Responses in Na^+^ and aldosterone for stepwise increase in salt intake (for parameter values, see main text). The level of salt intake starts at $k_{p}^{i}\;=\;1\cdot 10^{-5}$ mol/min in the lower functional space (dark gray). The light gray part illustrates the operational space. The letters *a–f* indicate steady‐state levels used in E. (E) The corresponding steady state relationship from the responses in D, where both the functional and operational areas are indicated. (F) Qualitative steady‐state relationship between body sodium and aldosterone, redrawn from Bonventre et al. (1982b).

From this description, we find from Figure 3A two possible controller candidates, that is, outflow controller 6 or outflow controller 8. The main difference between these two motifs is that sodium either activates the aldosterone degradation or inhibits the aldosterone synthesis, respectively. Both will, however, decrease aldosterone concentration at high plasma sodium concentration. Though, based on the fact that at low sodium level, aldosterone synthesis is stimulated (Garrett et al. 2012), the overall description fits an outflow controller 8. This is illustrated in Figure 7A, where $k_{p}^{i}$ represents the salt intake, and the corresponding model equations for the system are:

$$\begin{array}{c} \dot{{\text{Na}}^{+}}=k_{p}^{i}-V_{\max}^{{\mathrm{Na}}^{+}}\cdot \frac{{\text{Na}}^{+}}{K_{M}^{{\mathrm{Na}}^{+}}+{\text{Na}}^{+}}\cdot \frac{K_{I}^{\mathrm{Aldo}}}{K_{I}^{\mathrm{Aldo}}+\text{Aldo}}\\ \dot{\text{Aldo}}=k_{s}^{\mathrm{Aldo}}\cdot \frac{K_{I}^{{\mathrm{Na}}^{+}}}{K_{I}^{{\mathrm{Na}}^{+}}+{\text{Na}}^{+}}-V_{\max}^{\mathrm{Aldo}}\cdot \frac{\text{Aldo}}{K_{M}^{\mathrm{Aldo}}+\text{Aldo}} \end{array}$$

The World Health Organization presents different recommendations with respect to sodium intake for human adults (World Health Organization 2012), though 500 mg/day seems to be a recurring number for the lowest recommended intake, with an upper level of 2300 mg/day (U.S. Department of Health and Human Services and U.S. Department of Agriculture 2015; World Health Organization 2012). These levels correspond to $k_{p}^{i,\mathrm{low}}\;=\;1.5\cdot 10^{-5}$ mol/min and $k_{p}^{i,\mathrm{high}}\;=\;6.9\cdot 10^{-5}$ mol/min. Normal levels of body sodium is reported to lie between 0.135 and 0.145 mol/L (Garrett et al. 2012), and we therefore define ${\text{Na}}_{\mathrm{low}}^{+}\;=\;0.135$ mol/L and ${\text{Na}}_{\mathrm{high}}^{+}\;=\;0.145$ mol/L. Examples of reported levels of aldosterone varies between 0.19 nmol/L at high salt intake and 0.83 nM at low salt intake (Fischbach et al. 2009), and hence, we define Aldo_low_ = 0.19 nmol/L and Aldo_high_ = 0.83 nmol/L.

We start by considering the parameters in the sets $D_{A}\;=\;\left\{K_{M}^{{\mathrm{Na}}^{+}},V_{\max}^{{\mathrm{Na}}^{+}}\right\}$ and $S_{EA}\;=\;\left\{K_{I}^{\mathrm{Aldo}}\right\}$. Based on the operational space of high and low plasma sodium concentration, aldosterone concentration and salt intake, we find from Equations (13) and (14) that *β*_1,denom_>0 and *β*_2,num_<0. Thus, from Table 2 we find $K_{I}^{\mathrm{Aldo}}<4.96\cdot 10^{-3}$ nmol/L, and the solutions to $K_{M}^{{\mathrm{Na}}^{+}}$ and $V_{\max}^{{\mathrm{Na}}^{+}}$ as a function of $K_{I}^{\mathrm{Aldo}}$ are shown in Figure 7 *B*. As indicated, we select $K_{I}^{\mathrm{Aldo}}\;=\;4\cdot 10^{-3}$ nmol/L, and find $V_{\max}^{{\mathrm{Na}}^{+}}\;=\;0.06$ mol/min and $K_{M}^{{\mathrm{Na}}^{+}}\;=\;2.4$ mol/L.

Moving on to the parameters in the sets $D_{E}\;=\;\left\{k_{s}^{\mathrm{Aldo}},V_{\max}^{\mathrm{Aldo}},K_{M}^{E}\right\}$ and $S_{AE}\;=\;\left\{K_{I}^{{\mathrm{Na}}^{+}}\right\}$, we find from the operational area that the regulatory system satisfies Equation (19), that is,

$$\frac{{\text{Na}}_{\mathrm{high}}^{+}}{{\text{Na}}_{\mathrm{low}}^{+}}>\frac{{\text{Aldo}}_{\mathrm{high}}}{{\text{Aldo}}_{\mathrm{low}}}$$

Since this condition holds, it means that the value of the parameter $K_{I}^{{\mathrm{Na}}^{+}}$ does not influence the steady‐state properties of the system. In order to identify suitable values for the parameters in the set $D_{E}$, we consider first the synthesis rate of aldosterone, $j_{s}^{\mathrm{Aldo}}$, shown in Equation (27),

(27) $$j_{s}^{\mathrm{Aldo}}\;=\;k_{s}^{\mathrm{Aldo}}\cdot \frac{K_{I}^{{\mathrm{Na}}^{+}}}{K_{I}^{{\mathrm{Na}}^{+}}+{\text{Na}}^{+}}$$

This rate is in the literature found to be in the interval 0.10–0.15 mg/day (Meisenberg et al. 2017), corresponding to 0.19–0.29 nmol/min. We assume further that the average of this interval corresponds to sodium being at Na^+^ = 0.140 mol/L, and thus, we find $k_{s}^{\mathrm{Aldo}}\;=\;0.48$ nmol/min.^2^ Based on this value, Figure 7C illustrates how the values of $V_{\max}^{\mathrm{Aldo}}$ and $K_{M}^{E}$ depend on, the yet unspecified, value of $K_{I}^{{\mathrm{Na}}^{+}}$.

As the inhibitory signaling between sodium and aldosterone synthesis in the model represents several intermediate steps, there is no literature value available for $K_{I}^{{\mathrm{Na}}^{+}}$. We therefore choose a value corresponding to the average between the high and low sodium values, that is, $K_{I}^{{\mathrm{Na}}^{+}}\;=\;0.140$ mol/L, indicated with a vertical line in Figure 7C. Thus, we find $V_{\max}^{\mathrm{Aldo}}\;=\;25$ nmol/min and $K_{M}^{\mathrm{Aldo}}\;=\;9\cdot 10^{-3}$ nmol/L

Using these parameter values, Figure 7D shows the responses in Na^+^ and aldosterone for a stepwise increase in $k_{p}^{i}$ from a value in the lower functional space, throughout the operational space, and into the upper functional space. The light gray area represents the operational space.

The steady‐state relationship between Na^+^ and aldosterone corresponding to the different steady‐state levels in Figure 7D are shown as functional and operational areas in Figure 7E. As we see, the profile is similar to the qualitative sketch found in Bonventre et al. (1982b), redrawn in Figure 7F.

Finally, we calculate the value of the relaxing factor similar to Equation (26). The ratio of the high to low perturbation rate constant is 6.9·10^−5^/1.5·10^−5^ = 4.6, and below we see how the variation in the controlled variable Na^+^ (ratio 1.07) assists the controller (ratio 4.3) in obtaining a ratio of 4.6:

$$\begin{array}{cc} \frac{k_{p}^{\mathrm{in},\mathrm{high}}}{k_{p}^{\mathrm{in},\mathrm{low}}} & =\frac{g_{I}\left(K_{I}^{\mathrm{Aldo}},{\text{Aldo}}_{\mathrm{low}}\right)}{g_{I}\left(K_{I}^{\mathrm{Aldo}},{\text{Aldo}}_{\mathrm{high}}\right)}\cdot \frac{g_{a}\left(K_{M}^{\mathrm{Na}},{\text{Na}}_{\mathrm{high}}^{+}\right)}{g_{a}\left(K_{M}^{\mathrm{Na}},{\text{Na}}_{\mathrm{low}}^{+}\right)}\\ & =\frac{20.6\cdot 10^{-3}}{4.8\cdot 10^{-3}}\cdot \frac{5.7\cdot 10^{-2}}{5.33\cdot 10^{-2}}\\ & =4.3\cdot 1.07\\ & =4.6 \end{array}$$

To summarize, we have used experimental data for high and low levels of sodium, aldosterone and salt intake to parameterize a rheostatic model of the renal sodium regulatory system. Thus, related to the discussion regarding physiological setpoints, we argue for the existence of a variable setpoint for sodium.

Even though the model complexity is limited, this example illustrates how a two‐component controller motif can be constructed based on available steady‐state values. Thus, the signaling kinetic structure applied in the model and the parameter values identified can serve as a base for comparison in the development of more complex models of body sodium regulation.

## Conclusion

We have in this paper introduced and examined a new facet into the puzzle and discourse on the notion of setpoints in physiology. Based on our previously published homeostatic controller motifs, we have identified plausible mechanisms behind the existence of a variable setpoint, which share similarities with the concept of rheostasis which again describes regulation around shifting setpoints (Mrosovsky 1990).

One of the aspects of a variable setpoint is that the setpoint depends on the level of the manipulated variable, that is, the concentration of the species that performs regulatory actions on the compensatory inflow or outflow of the controlled variable. Moreover, the level of the manipulated variable is again dependent on the level of disturbance (or perturbation), which is the driving force behind a varying setpoint. Our explanation behind a variable setpoint is therefore a combination of the two alternatives presented by Woods and Ramsay (2007), as they stated the following: “The point is that an interpretational complexity arises in studies on homeostasis because an observed change in a regulated variable can result from a forced deviation away from its defended value by an externally arising disturbance or else from a rheostatic adjustment of the value to a new defended level.”

Since the high and low levels of (1) the perturbation $k_{p}^{i/o}$, (2) the manipulated variable *E* and (3) the controlled variable *A* are related, we have further defined an operational region of the controller motif. This operational region imposes constraints on the different motif parameters, and we have identified conditions on the signaling kinetics parameters between *A* and *E*, that is, the activation and inhibition constants.

In effect, our approach comprises both the fixed setpoint approach (homeostatic system) and the variable setpoint approach (rheostatic system) in a single formulation. In order to define a homeostatic system, it is only a matter of defining the variability of the controlled (or regulated) variable. Hence, an approximate fixed (homeostatic) setpoint is achieved by specifying *A*_high_ = *A*_low_+*ε*, where *ε* is an adequate small number. In this context, *ε* will reflect what we defined as the accuracy *α* in (Drengstig et al. 2012a) to describe deviation from the fixed setpoint.

We have further shown that the notion of a variable setpoint is indeed a relaxing component in that the variation in the controlled variable (being the rheostatic setpoint) reduce the effort needed from the controller species to counteract the effect of the disturbance. Hence, from an evolutionary point of view, the rheostatic setpoint represents a trade‐off between energy savings and possible disadvantages from variations in the regulated variable. This represents an optimization problem, and is a topic for ongoing research.

## Conflict of Interest

None declared.

## Notes

1 Although a better term is *inaccuracy* as pointed out in Thorsen (2015).

2 Note that this value represents the controller gain as shown in Table 1.
